# Time-to-event studies: how robust are inferences when the assumption of independent censoring is in doubt?

**DOI:** 10.1186/1745-6215-16-S2-P138

**Published:** 2015-11-16

**Authors:** Martin Law, Dan Jackson

**Affiliations:** 1 Medical Research Council Biostatistics Unit, Cambridge, UK

## 

The Cox proportional hazards model is often used to analyse time-to-event data. In many patients, the event time is unknown, either due to dropout or study end. This is known as censoring. When using the Cox proportional hazards model, it is assumed that censoring is independent of event time. However, this is an untestable, often implausible assumption; it may be that censoring is associated with a change in the hazard of an event.

Our interest is in examining departures from this assumption, as a sensitivity analysis. One recently-proposed approach involves treating event time data of censored patients as missing, adjusting the hazards of those patients, and using multiple imputation to impute the missing event times.

How the hazard changes after censoring is determined by a user-defined sensitivity parameter. A positive value indicates an increase in hazard (harmful effect), while a negative value indicates a decrease (protective effect). The most basic change in hazard is a step change applied to all censored patients. However, the change may be time-dependent, or applied to a subset of censored patients based on their covariates or strata.

By examining how parameter estimates change as this sensitivity parameter is varied, we see how robust our analysis is to the assumption of independent censoring.

We apply this approach for the first time to example data containing stratified data, in an area for which there is prior evidence that censoring is associated with event hazard. We relax the independent censoring assumption, and show how the parameter estimates change.

